# Understanding the Reaction-to-Fire Properties of Biomass and Their Respective Biochars

**DOI:** 10.1021/acsomega.5c01207

**Published:** 2025-09-22

**Authors:** Elif Kaynak, Vigneshwaran Shanmugam, Jacob Johansson, Kesavarao Sykam, Sidique Gawusu, Linda Makovická Osvaldová, Rhoda Afriyie Mensah, Lin Jiang, Emre Uraz, Chia-feng Lin, Oisik Das

**Affiliations:** 1 Department of Civil, Environmental and Natural Resources Engineering, 5185 Luleå University of Technology, Luleå 97187, Sweden; 2 Department of Chemistry, Faculty of Science and Technology, 510137 (IcfaiTech) ICFAI Foundation for Higher Education, Hyderabad 501203, India; 3 Whiting School of Engineering, 1466 Johns Hopkins University, Baltimore, Maryland 21218, United States; 4 Department of Fire Engineering, University of Zilina, Univerzitná 8215/1, Žilina 010 26, Slovakia; 5 School of Mechanical Engineering, 12436 Nanjing University of Science and Technology, Nanjing 210094, China; 6 Chemical Engineering Department, 522675 Eskişehir Technical University, Eskişehir 26555, Turkey; 7 Wood Science and Engineering, Department of Engineering Sciences and Mathematics, Luleå University of Technology, Skellefteå 93187, Sweden

## Abstract

Waste biomass presents both environmental challenges and opportunities for sustainable innovation. Converting biomass into biochar provides an environmentally sustainable method for waste valorization, with biochar demonstrating considerable potential for enhancing fire safety in various material applications. This study examines the fire performance of various biomass feedstocks (reed pellets, olive pits, wood chips, rubber waste) and their corresponding biochars, produced at 700 °C, using cone calorimeter tests. The biomass samples exhibited peak heat release rates (PHRR) ranging from 200 to 600 kW/m^2^, whereas the PHRR of their biochars was significantly lower, ranging from 20 to 35 kW/m^2^. Biochar derived from lignocellulosic biomass demonstrated more prolonged combustion behavior, with higher PHRR values, whereas rubber waste biochar exhibited the lowest PHRR (21.3 kW/m^2^) and total heat release (THR) (4.1 MJ/m^2^), highlighting its enhanced fire safety compared to its biomass counterpart. The composition of the parent biomass plays a crucial role in determining the biochar’s fire performance. These findings offer valuable insights into the selection of biochar for various applications, particularly as a filler in flame-retardant composites.

## Graphical abstract

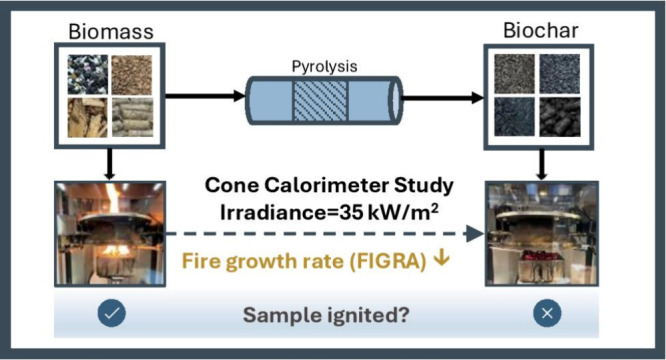

## Introduction

In order to attain sustainable development goals, valorization of biomass wastes is gaining momentum, both in academia and the industry. Numerous wastes, such as sewage sludge, sawdust, sugar cane bagasse, coconut shells, and rice husk, are classified as biomass. Lignocellulosic biomass, accounting for the majority of the biomass wastes, is made up of a mixture of the most commonly occurring natural polymers in the form of lignin, cellulose, and hemicellulose.^1^ One of the desired products from biomass valorization is biochar, which is the carbonaceous solid product of thermo-chemical conversion under limited oxygen or inert conditions. Biochar is favored over the other thermo-chemical conversion products, such as bio-oil and syngas, because it can be directly used in a variety of applications without further refinement. Biochar can be engineered to possess unique properties such as high carbon content, thermal stability, high surface area, and high particle hardness/modulus.^2,3^ Biochar can be produced mainly through three methods: pyrolysis, hydrothermal carbonization, and gasification (biochar is a byproduct having a very low yield).^4,5^ When biomass undergoes thermo-chemical conversion under high temperature (>500 °C) and long residence time (ca. >1 h), the volatiles responsible for combustion (e.g., aliphatic groups) are removed leaving behind a porous, carbonaceous, thermally stable biochar having strong C–C covalent bonds. However, the pyrolysis of biomass is a complex process in which the decomposition of its main components, i.e., hemicellulose, cellulose, and lignin, occurs over overlapping temperature intervals.^3,6^ The first stage, below 200 °C, is typically attributed to the evaporation of moisture. Hemicellulose mainly decomposes between 220 and 315 °C, followed by cellulose, which primarily decomposes between 315 and 400 °C. Lignin undergoes relatively slow decomposition up to 900 °C. Numerous seminal research has been conducted to comprehend the thermo-chemical pathway for the formation of biochar.^7–9^ The process regarding lignocellulosic biomass can be summarized as the chemical bonds among the biopolymers of wood (cellulose, hemicellulose, and lignin) begin to disassemble when subjected to high temperature. These reactions, coined as “primary pyrolysis reactions”, may produce products that are not volatile enough and hence, can further react, breaking internal bonds to form products, which are volatile or may condense or polymerize to form biochar. The resultant volatiles may again react in the pores of the biomass particle, which are called “secondary intraparticle reactions”. These secondary intraparticle reactions may happen homogeneously in the gas phase or heterogeneously with the produced biochar and partially decomposed biomass. The pyrolysis vapors are generally generated on the biomass cell wall, which diffuse through a very thin layer of biochar formed into the hollow section of the biomass particle and then transfer out of the particle to the bulk of the gas. The volatiles after being ejected out the biomass particle may still participate in homogeneous and heterogeneous reactions, which are called “extra-particle secondary reactions”. Both the intraparticle secondary and extra-particle secondary reactions may cause significant changes in the yield of products (i.e., biochar, bio-oil, and syngas) obtained.

Although the formation mechanisms of biochar via primary and secondary pyrolysis reactions have been widely studied, the fire behavior of biochar especially in comparison to its parent biomass remains underexplored. Notably, it was observed by Das et al. that when biochar is made at a temperature of 900 °C, all the volatiles escape leaving behind a carbon skeleton having aromatic structure.^10^ The absence of volatiles and strong covalent bonds among the carbon atoms of the biochar can bestow fire-safety properties wherein the biochar can resistant ignition at a heat flux of 50 kw/m^2^ and have particle hardness of 4.3 GPa.^10^ Thus, this unique property of biochar can be capitalized to develop composites having biochar, which can simultaneously impart fire safety and improve mechanical properties through an additive effect.

Growing interest in biochar stems from its multifunctional advantages ranging from mechanical reinforcement to fire resistance as well as its potential to reduce environmental impact in applications such as polymers and concrete. In polymer composites, incorporating up to 35 wt % wood waste biochar into polypropylene significantly enhanced tensile and flexural strength, while also reducing the peak heat release rate through the formation of a compact, insulating char layer.^11^ Carbon black, pine bark, and gluten biochars improved the modulus of wheat gluten composites, with gluten biochar providing the highest stiffness and water resistance, which are critical features for moisture-sensitive applications, due to favorable chemical interactions and intrinsic hardness.^12^ In concrete, using fine or coarse biochar (10–20 wt %) as partial cement or aggregate replacement improved fire resistance (PHRR < 40 kW/m^2^) and lowered CO_2_ emissions.^13^ Another study showed that incorporating just 3 wt % biochar enhanced compressive strength at both ambient and elevated temperatures (up to 700 °C), indicating improved thermal stability and postfire structural retention.^14^ Several studies have determined the fire behavior of biochar-added composites;^15–17^ however, there remains a dearth of studies that have investigated the innate reaction-to-fire properties of biochar. This presents an opportunity to gain insight into the transition of reaction-to-fire properties from the parent biomass to its corresponding biochar. It is known from the literature that the properties of biochar are greatly influenced by the production method, pyrolysis temperature, and the type of biomass feedstock.^18,19^ However, the influence of these factors on the fire performance of biochar, particularly in relation to the composition of the original biomass, has not been thoroughly investigated. This study addresses this gap by investigating the reaction-to-fire properties of various biomass feedstocks and their corresponding biochars. Moreover, a novel approach was adopted to identify the biopolymer composition of biomass from thermogravimetric analysis (TGA) data, providing a more detailed understanding of how biomass composition affects fire behavior. The increasing use of biomass across various industries also raises concerns regarding potential fire hazards during its handling and storage. Hence, it is important to explore the reaction-to-fire properties of the various biomass feedstocks and their corresponding biochars.

Cone calorimetry is a widely accepted method for evaluating the ignitability and heat release characteristics of materials at the small scale.^20^ It has also been used to generate data for simulating fire development in full-scale or real-world scenarios although its ability to replicate under-ventilated fire conditions may be limited.^21,22^ Nevertheless, it remains a powerful tool for the comparative evaluation of the fire performance of materials. In this study, a cone calorimeter was used to analyze how fire performance evolves from raw biomass (olive pits, wood chips, reed pellets, and rubber waste) to the corresponding biochars, and how different biomass sources influence the fire properties of the resulting biochars. Although not lignocellulosic in nature, rubber waste is referred to as “biomass” and its carbonization product as “biochar” throughout this article to maintain terminological consistency. Key fire response parameters including time to ignition (TTI), peak heat release rate (PHRR), total heat release (THR), maximum average rate of heat emission (MARHE), time to peak heat release rate (TTPHRR), mass loss rate (MLR), fire growth rate (FIGRA), and fire performance index (FPI) were evaluated by the cone calorimeter under a uniform external heat flux of 50 kW/m^2^ to simulate fully developed fire conditions.

## Results and Discussion

### Time to Ignition and Time to Peak Heat Release Rate

The values of TTI, PHRR, TTPHRR, THR, and MARHE were measured by the CC, while the FIGRA and FPI were calculated from the CC’s output (Table 1). TTI is a key indicator of a material’s ease of ignition and its flammability characteristics.^23^ Reed biomass achieved the highest TTI of 89 ± 3 s, while rubber waste achieved a TTI of 13 ± 2 s. The relatively higher TTI of the lignocellulosic biomass can be attributed to the presence of relatively stable aromatic networks. Similarly, the time needed to achieve the PHRR (TTPHRR) was the shortest for rubber waste at 22 ± 3 s, indicating that it reached the PHRR within 9 s after ignition. The reed biomass exhibited the longest TTPHRR (Table 1) at 230 ± 3 s, with the time to reach the PHRR after ignition approximately 140 s. Due to the low volatile content of biochar following pyrolysis, the TTI could not be measured in the cone calorimeter tests. Figure 1 depicts the nonignitability and glowing behavior of the biochars through digital pictures taken during the CC tests. By means of TTPHRR, a higher value indicates slow burning characteristics. TTPHRR values for rubber biochar, olive pits biochar, wood chips biochar, and reed biochar are 35 ± 6, 359 ± 52, 134 ± 3, and 243 ± 5 s, respectively (Table 1).

**Figure 1:**
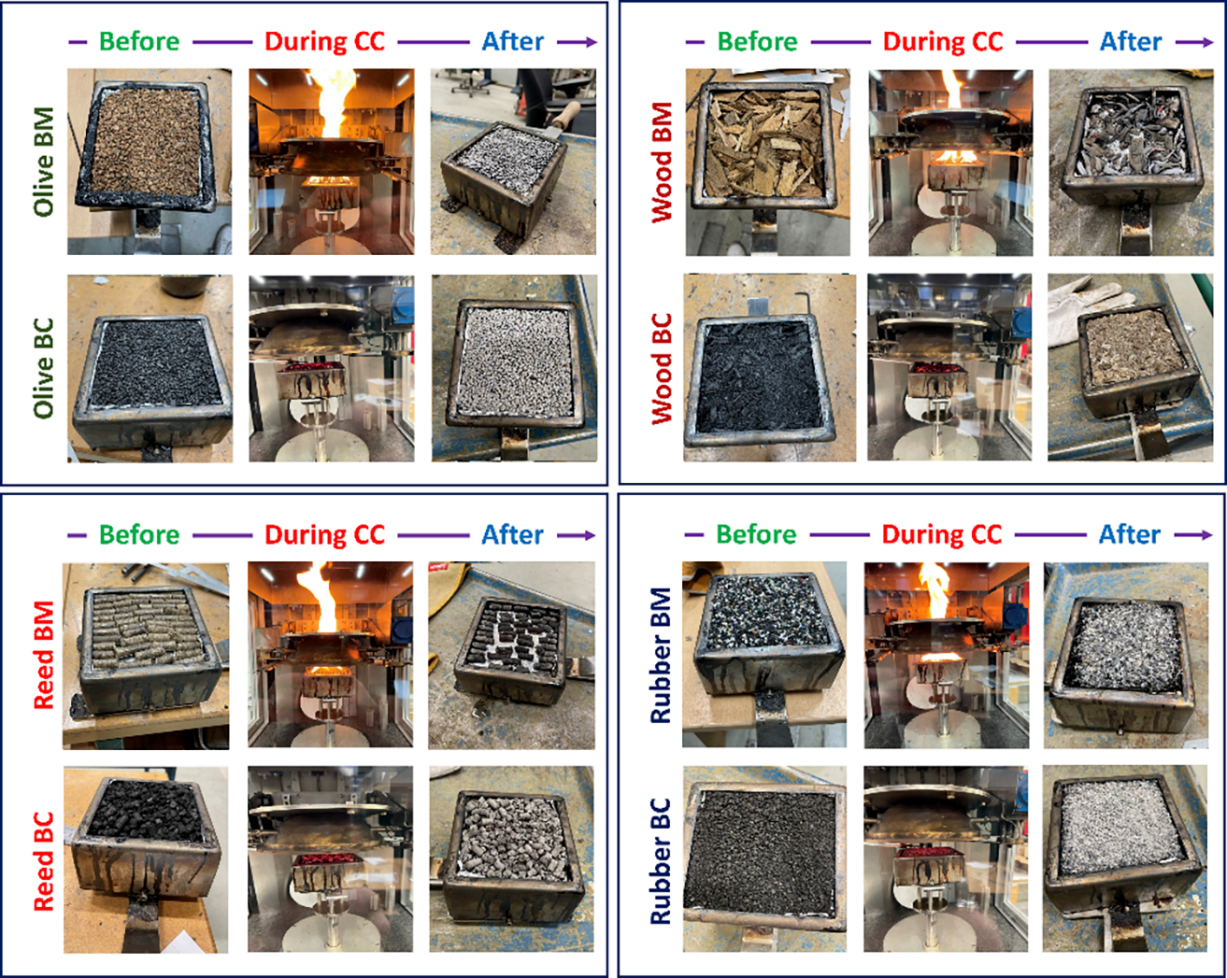
Digital photographs of the various biomass (BM) feedstocks and their respective biochars (BC) before and after cone calorimetry (CC) experiments.

**Table 1:** Cone Calorimeter Data of Various Biomass Feedstocks and Respective Biochar

	reaction-to-fire properties						
sample	PHRR [kW/m^2^]	TTPHRR [s]	**THR** **[MJ/m** **^2^** **]**	TTI [s]	FIGRA [kW/m^2^ s]	FPI [m^2^·s/kW]	MARHE [kW/m^2^]
rubber waste—biomass	417.3 ± 28.9	22 ± 3	61.6 ± 1.3	13 ± 2	19.51 ± 3.98	0.03 ± 0.01	321.5 ± 9.5
rubber waste—biochar	21.3 ± 0.6	35 ± 6	4.1 ± 0.3	NA	0.63 ± 0.08	NA	16.5 ± 0.5
olive pits—biomass	597.7 ± 36.1	85 ± 9	39.8 ± 0	52 ± 5	7.19 ± 1.15	0.09 ± 0.01	251.0 ± 19.0
olive pits—biochar	30.6 ± 0.6	359 ± 52	20.6 ± 0.1	NA	0.09 ± 0.01	NA	23.0 ± 0
wood chips—biomass	232.7 ± 0.2	41 ± 3	34.6 ± 0.3	35 ± 2	5.77 ± 0.25	0.15 ± 0.01	156.0 ± 2.0
wood chips—biochar	29.7 ± 0.7	134 ± 3	21.7 ± 0.2	NA	0.22 ± 0	NA	24.5 ± 0.5
reed—biomass	366.1 ± 4.0	230 ± 3	73.5 ± 1.1	89 ± 3	1.59 ± 0.04	0.24 ± 0.01	165.0 ± 3.0
reed—biochar	28.6 ± 0.7	243 ± 5	19.4 ± 0.8	NA	0.12 ± 0	NA	22 ± 1 ± 0.4

As shown in Figure 2, reed pellets exhibited the highest TTPHRR among the biomass samples, whereas olive pit biochar exhibited the highest TTPHRR among the biochars. In comparison to their respective biomass, it can be stated that all biochars have shown a delay in TTPHRR.

**Figure 2:**
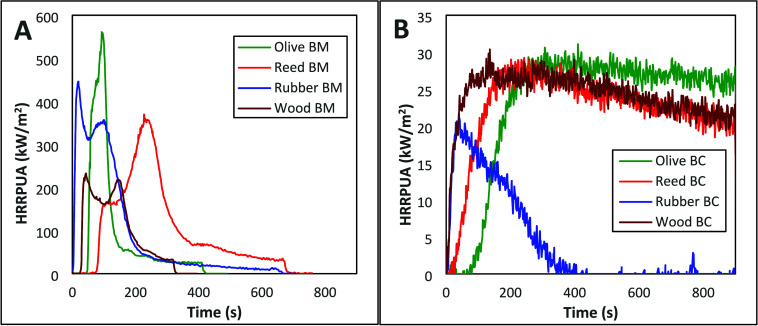
Plots of HRR vs time for (A) biomass (BM) feedstocks and (B) respective biochars (BC).

### Heat Release Rate (HRR)

Figure 2 shows the heat release rate per unit area (HRRPUA) vs time of the biomass and biochar samples used in this study. The HRRPUA vs time curves for the lignocellulosic biochar followed a similar pattern, i.e., they attain their peak and thereafter decline slightly. However, the HRRPUA vs time curves exhibit distinct patterns across different lignocellulosic biomass. Olive pit biomass showed a single peak, whereas reed pellets exhibited a shoulder before the main peak, indicating charring; however, this charring was not sufficient to protect the underlying material. A low value of PHRR indicates a safer material regarding fire safety.^24^ PHRR values of 417.3 ± 28.9, 597.7 ± 36.1, 232.7 ± 0.2, and 366.1 ± 4.0 kW/m^2^ were recorded for rubber waste, olive pits, wood chips, and reed biomass, respectively, whereas the PHRR values of the respective biochars were 21.3 ± 0.6, 30.6 ± 0.6, 29.7 ± 0.7, and 28.6 ± 0.7 kW/m^2^, respectively (Table 1). The wood chips exhibit the lowest PHRR among the lignocellulosic biomass studied.^30^ The rubber waste biochar shows the lowest PHRR among the biochars, approximately 70–75% of that of lignocellulosic biochars.

### Mass Loss Rate (MLR) and Correlation with HRR

The HRR and MLR patterns of the biomass are shown in Figures 2A and 3A, respectively. Similar mass loss and HRR patterns were observed for all of the biomass. The PHRR and the respective maximum MLR occurred on the same timeline. The mass loss patterns of the biochars of the olive pits, reed, rubber, and wood suggest the maximum MLR happened beyond 100 s. However, the low MLR of rubber biochar was clearly distinguished from the rest as can be seen in Figure 3B.

**Figure 3:**
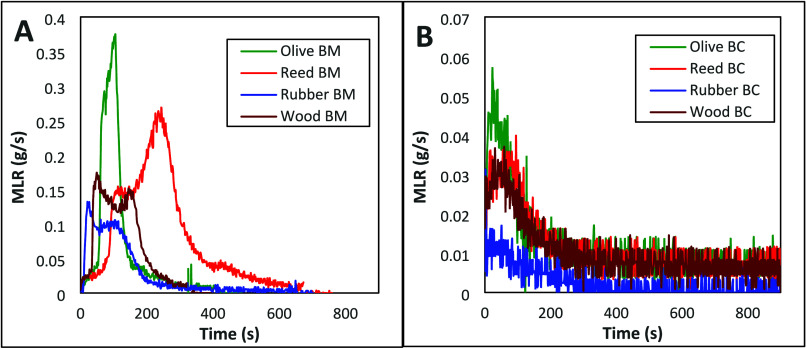
Representative diagram of MLR vs time plots of (A) biomass (BM) feedstocks and (B) respective biochars (BC).

### Total Heat Release (THR) and Fire Growth Rate (FIGRA)

The representative THR profiles of the biomass feedstocks and respective biochars are depicted in Figure 4. It is noted that the wood biomass exhibited the lowest THR of 34.6 ± 0.3 MJ/m^2^ whereas the reed biomass showed the highest THR (73.5 ± 1.1 MJ/m^2^). The biochar derived from rubber waste was by far the lowest regarding THR (18–21% lower than lignocellulosic biochars) with a THR of 4.1 ± 0.3 MJ/m^2^.

**Figure 4:**
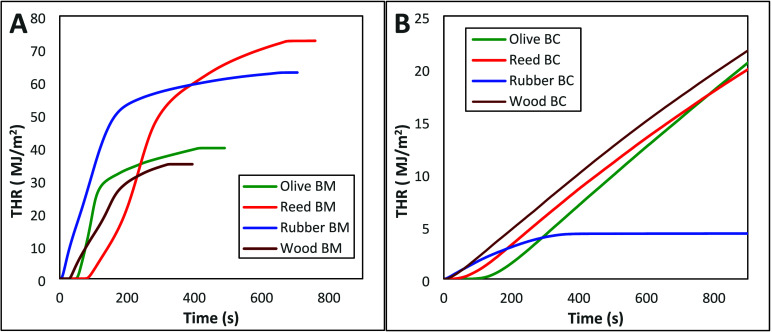
Representative diagram of THR vs time plots of (A) biomass feedstocks and (B) respective biochar.

In general, a low MARHE value indicates a low potential for fire growth.^25^ With a MARHE of 156.0 ± 2.0 kW/m^2^, wood chips were safer than reed pellets, which exhibited a MARHE of 165.0 ± 3 kW/m^2^ (Table 1). No significant difference was observed among MARHE values of the biochars, which ranged from 16 to 25 kW/m^2^. Lower FIGRA values indicate a slow fire growth rate, hence safer materials are expected to have lower FIGRA values. As presented in Table 1, in terms of FIGRA the reed biomass performed the best with a value of 1.59 ± 0.04 kW/m^2^·s while the biochar made from olive pits exhibited the lowest value of 0.09 ± 0.01 kW/m^2^·s. The FPI parameter, on the other hand, should be high to indicate a higher fire safety rank. Since it is calculated based on time to ignition (eq 2), it can only be applied to the biomass samples. It can be concluded from the data (Table 1) that reed biomass performed the best with an FPI value of 0.24 ± 0.01 m^2^·s/kW whereas rubber performed the worst with an FPI of 0.03 ± 0.01 m^2^·s/kW.

### Comparative Performance of Biomass and Biochar

It is difficult to choose the optimal biomass or biochar based on these flammability factors since no single system has shown the greatest performance for all reaction-to-fire parameters. Hence, Table 2 was prepared to facilitate a comparison by summarizing the best- and worst-performing samples across the various reaction-to-fire parameters tested.

**Table 2:** Comparative Analysis in Terms of Reaction-to-Fire Properties of the Biomass and Respective Biochar

	biomass		biochar	
reaction-to-fire parameters	at best	at least	at best	at least
PHRR [kW/m^2^]	wood chips	olive pits	rubber	olive pits
TTPHRR [s]	reed pellets	rubber	olive pits	rubber
THR [MJ/m^2^]	wood chips	reed pellets	rubber	wood chips
TTI [s]	reed pellets	rubber	NA	NA
FIGRA [kW/m^2^s]	reed pellets	rubber	olive pits	rubber
FPI [m^2^s/kW]	reed pellets	rubber	NA	NA
MARHE [kW/m^2^]	wood chips	rubber	rubber	wood chips

As shown in Table 2, the biomass from rubber waste performed the least in five out of seven parameters and is the least in terms of fire safety, whereas the reed pellets performed the best in four out of seven parameters (although having the highest THR). However, when converted into biochar as presented in Table 2, the rubber waste showed significant improvement and performed the best in three parameters, namely, PHRR, THR, and MARHE. While the incorporation of biochars derived from lignocellulosic biomass has been widely reported to enhance the fire safety of thermoplastic composites such as polypropylene and high-density polyethylene,^11,26^ to the best of the authors’ knowledge, the effects of biochar derived from rubber waste in fire retardancy of such composites have not yet been investigated. The biochar derived from olive pits also improved in its reaction-to-fire properties, having performed best in both TTPHRR and FIGRA, although it still exhibited the highest PHRR in both biomass and biochar. The biochar of wood chips performed the worst in two parameters, THR and MARHE, despite having the lowest THR among the biomass samples.

During combustion, lignocellulosic biomass forms a char layer that promotes a barrier effect, slowing down heat and mass mass transfer.^27^ The second peak in the heat release rate (HRR) is often attributed to the cracking of this protective char layer.^28^ The chemical composition of the biomass, particularly the relative contents of cellulose and lignin, strongly influences char formation and overall combustion behavior. As shown in Table 3, the contents of hemicellulose, lignin, and cellulose were similar among the studied biomass samples, with no substantial differences observed. In general, biomass with higher lignin content tends to exhibit lower PHRR, as lignin decomposes more slowly over a wider temperature range and promotes stable char formation.^27^ Wood chips having the highest cellulose (36 wt %) and lignin (39 wt %) content exhibited the lowest PHRR (232.7 ± 0.2 kW/m^2^) and THR (34.6 ± 0.3 kW/m^2^). Since the variation in PHRR and THR cannot be fully explained by the lignin, cellulose, and hemicellulose contents alone, it is worth noting that other factors, such as the content of extractives and trace elements, may also influence fire response. For instance, the relatively high PHRR of olive pit biomass could be related to the presence of residual oil.^29^

Although all biochar from lignocellulosic biomass performed similarly in terms of PHHR, THR, and MARHE, noticeable differences were observed in TTPHRR and FIGRA. Since FIGRA is calculated based on TTPHRR, the only independent parameter (TTPHRR) that shows significant differences between the biochar samples is TTPHRR. Olive pit biochar exhibited the highest TTPHRR, while wood chip biochar showed the lowest. However, since combustion properties are strongly influenced by the physical and chemical characteristics of the resulting biochar, these trends cannot be solely attributed to the cellulose and lignin contents of the parent biomass (Table 3).

**Table 3:** Cellulose, Hemicellulose, and Lignin Content of the Biomass Species on a Dry Basis

	computed values (thermal analysis)			literature values (chemical analysis)			
sample	cellulose (wt %)	hemicellulose (wt %)	lignin (wt %)	cellulose (wt %)	hemicellulose (wt %)	lignin (wt %)	ref
olive	32	30	38	31	26	36	^30^
wood	36	26	39	31	21	38	^31^
reed	32	33	35	16	25	27	^32^

The biochar derived from rubber waste exhibited a distinctly different HRR profile compared to those from lignocellulosic biomass. It outperformed the rest of the biochars from lignocellulosic biomass, in terms of reaction-to-fire properties excluding TTPHRR and FIGRA (which is based on TTPHRR). This might imply that the biochar made from rubber waste forms chemical compounds and bonds different from those formed by the polycondensation of cellulose and lignin.

Rubber waste also exhibited a markedly lower TTI (13 ± 2 s) compared to that of biomass such as reed pellets, which had the highest TTI (89 ± 3 s) among the tested samples. Although TTI is commonly attributed to the volatile content,^33^ it is understood that the nature of the volatiles also plays a critical role in the ease of ignition (Table 4).^34,35^ The low TTI of rubber can be ascribed to its high carbon (84.1%) and low oxygen content (0.8%), whereas oxygenated functional groups in biomass promote dehydration reactions during the initial combustion stages, releasing water. The high TTI of reed pellets may be attributed to their compact physical structure, which results in lower porosity and higher thermal conductivity, thereby delaying surface temperature rise and ignition.^36^

**Table 4:** Chemical Composition of Various Feedstock

sample	physical form	volatile (wt %)	C (wt %)	H (wt %)	N (wt %)	O (wt %)	ref
rubber	granules	61.3	84.1	7.3	0.3	0.8	^41^
olive	granules	80.9	52.2	7.5	0.1	40.1	^37^
wood	chips	86.6	47.7	5.7	0.1	46.5	^38^
reed	pellets	79.3	40.2	5.6	0.6	47.2	^32^

## Conclusions

In a nutshell, the final reaction-to-fire properties of various locally available biomass feedstocks such as reed pellets, olive pits, wood chips, rubber waste, and their respective biochars produced at 700 °C were studied by a cone calorimeter. Biomass samples exhibited considerable combustibility, with high peak heat release rates (PHRR) ranging from 232.7 to 597.7 kW/m^2^ and total heat release (THR) values between 34.6 and 73.5 MJ/m^2^. In contrast, their corresponding biochars showed significantly improved fire behavior. In particular, rubber-waste-derived biochar outperformed others with the lowest peak heat release rate (21.3 ± 0.6 kW/m^2^) and total heat release (4.1 ± 0.3 MJ/m^2^). Unlike lignocellulosic biochars, which exhibited prolonged burning and broader HRR profiles, rubber waste biochar showed a relatively sharp HRR peak indicating a distinct combustion mechanism due to its unique chemical composition. This underscores the significance of biomass origin for the development of biochar-based materials with enhanced fire safety.

## Experimental Section

### Materials Used

The biochars in this study are produced by Novo Carbo, Germany, via pyrolysis at 700 °C. The cellulose, hemicellulose, and lignin contents of the biomass were determined from thermogravimetric analysis (TGA) data following a previously reported procedure^39^ with minor modifications. The modified procedure, along with the calculated kinetic parameters and the corresponding model fits to the experimental DTG curves for each biomass sample, is presented in the Supporting Information (SI).

### Cone Calorimeter Test

The reaction-to-fire properties of the biomass and their respective biochars were evaluated by using a TCC 918 Cone Calorimeter (CC) (Netzsch Taurus GmbH, Weimar, Germany) according to the manufacturer’s specifications as well as the standard ISO 5660-1:2015. The tests were conducted under an external heat flux of 35 kW/m^2^. Generally, 10 cm × 10 cm block samples are tested in a cone calorimeter according to ISO 5660. However, due to the nature of the samples in this study (powders, chips and pellets), approximately 50 g of each was evenly distributed on aluminum foil, as described elsewhere.^40,41^ For the biochar samples, specifically, the layer thickness was set to approximately 5 mm. Due to the inherent variation in the form of the biomass samples, the thickness was maintained between 5 and 10 mm. The tests were performed in duplicate, and the average results were reported. FIGRA and FPI were determined by using the cone calorimeter data.

FIGRA is one way to evaluate the test data for a product’s fire classification. FIGRA (kW/m^2^·s) is defined as the growth rate of the burning intensity and calculated by dividing the peak heat release rate by the TTPHRR according to eq 1.^42^

$$\mathrm{FIGRA}=\frac{\mathrm{PHRR}}{\mathrm{TTPHRR}}$$ 1

FPI can give an overall assessment of a material’s fire safety. A higher value of the FPI indicates a higher safety rank. Using the test results from the CC, FPI (m^2^s/kW) can be calculated as the ratio between the TTI and the PHRR as seen in eq 2.^43^

$$\mathrm{FPI}=\frac{\mathrm{TTI}}{\mathrm{PHRR}}$$ 2

## Supplementary Material



## Notes

Experimental method for thermogravimetric analysis; derivation and application of the pseudo component model for kinetic calculations; kinetic parameters and pseudocomponent compositions of biomass; and TGA/DTG curves and corresponding model fits for biomass (PDF)
