# Supplementary material for: Understanding the Reaction-to-Fire Properties of Biomass and Their Respective Biochars
Source: ACS Omega. 2025 Sep 22;10(39):44895–902. doi: 10.1021/acsomega.5c01207 (PMC12508914; doi:10.1021/acsomega.5c01207)
Supplement: Supplementary file 1 [file ao5c01207_si_001.pdf]

Supporting Information for

Understanding the reaction-to-fire properties of biomass and their respective biochars

Elif Kaynak[1,ǂ*], Vigneshwaran Shanmugam[1,ǂ], Jacob Johansson[1], Kesavarao Sykam[2], Sidique Gawusu[3], Linda Makovická Osvaldová[4], Rhoda Afriyie Mensah[1], Lin Jiang[5], Emre Uraz[6], Chia-feng Lin[7], Oisik Das[1,*]

[1]*Department of Civil, Environmental and Natural Resources Engineering, Luleå University of Technology, 97187 Luleå, Sweden.*

[2]*Department of Chemistry, Faculty of Science and Technology, (IcfaiTech), ICFAI Foundation for Higher Education, Hyderabad-501203, India*

[3]*Whiting School of Engineering, Johns Hopkins University, Baltimore, MD 21218, United States.*

[4]*Department of Fire Engineering, University of Zilina, Univerzitná 8215/1, 010 26 Žilina, Slovakia.*

[5]*School of Mechanical Engineering, Nanjing University of Science and Technology, Nanjing 210094, China*

[6] *Chemical Engineering Department, Eskişehir Technical University, Eskişehir 26555, Turkey*

[7]*Wood Science and Engineering, Department of Engineering Sciences and Mathematics, Luleå University of Technology, 93187, Skellefteå, Sweden*

[ǂ]These authors contributed equally to this manuscript.

**Corresponding authors:* oisik.das@ltu.se *(O.D.)* elif.kaynak@associated.ltu.se *(E.K.)*

Method

A pseudocomponent kinetic model is used to estimate lignocellulosic fractions in the samples. The derivative thermogravimetric (DTG) curve obtained from a thermoanalytical curve of a thermogravimetric (TG) analysis usually has 2 distinct peaks. The first one represents the removal of moisture with a peak occurring before 100°C and usually ending near 150°C. The next one is the decomposition of cellulose yielding a peak between 300-340°C and ending sharply just before 400°C. Usually, decomposition of hemicellulose fractions can be observed around cellulose as a distinct peak or as a shoulder on the curve of cellulose decomposition reaction. These distinct peaks help to place decomposition curves for components that are created using the two principle kinetic parameters, activation energy and rate constant (frequency factor) and eventually determining their fraction.

Thermogravimetric analyses (TGA) (PerkinElmer TGA 4000, Waltham, MA, USA) were conducted between 30°C and 840°C at a heating rate of 5K/min under a nitrogen flow of 100mL/min.

In conventional kinetic analysis, multiple thermoanalytical curves are obtained at different heating rates and the kinetic parameters can be determined using different approaches. To determine the composition of a sample, however, the application of DTG curve is more useful since the curve itself is more representative of trends and is easier to get meaningful results in curve fitting processes.

Conversion is used instead of weight for kinetic studies. Conversion (α) is calculated as follows:

$$\alpha_i = \frac{m_0 - m_i}{m_0 - m_\infty} \quad \text{(Eq. S1)}$$

Here, $m_0$is the initial mass, m_∞ is the final mass and m_i denotes the mass at a discrete time. After the mass data is converted to conversion data, the DTG curve is obtained by differentiating the conversion data against temperature.

Assuming the biomass is composed of only cellulose, hemicellulose, lignin and water, the overall mass balance for components and the biomass during the decomposition would be as follows:

$$\frac{d\alpha}{dt} = \frac{d\alpha_W}{dt} + \frac{d\alpha_C}{dt} + \frac{d\alpha_H}{dt} + \frac{d\alpha_L}{dt} \quad \text{(Eq. S2)}$$

The rate is generally expressed as a product of a conversion-dependent function and a temperature-dependent function, assuming that the reaction rate depends solely on these two variables:

$$\frac{d\alpha}{dt} = f(\alpha)k(T) \qquad \text{(Eq. S3)}$$

While the conversion function can be a model or an expression, the rate function is the Arrhenius equation:

$$k(T) = Aexp(-E/RT) \qquad \text{(Eq. S4)}$$

In the Arrhenius equation, E denotes the activation energy (kJ/mol) and R (J/mol·K) is the gas constant. Combining Eq. S3 and Eq. S4 and introducing the heating rate, β (K/min), to convert the equation from time domain to temperature domain the following general statement is obtained.

$$\frac{d\alpha}{f(\alpha)} = \frac{A}{\beta}\exp(-E/RT)dT \qquad \text{(Eq. S5)}$$

Assuming the reaction is a first-order reaction, Eq. S5 will become:

$$\frac{d\alpha}{\alpha} = \frac{A}{\beta}\exp(-E/RT)dT \qquad \text{(Eq. S6)}$$

The solution of Eq. S6 involves the temperature integral which has no analytical solution. Integrating Eq. S6 gives the following equation that can be used for each component.

$$\alpha_j = \alpha_{j,0}exp\left\{-\frac{A_j}{\beta}\int_{T_0}^{T}\exp(\frac{-E_j}{RT})\right\} \qquad \text{(Eq. S7)}$$

At any temperature, the mass balance given in Eq. S2 should hold. This requires the solution of temperature integral at each temperature and using Eq. S7 for each component in Eq. S2.

Root Mean Squared Error (RMSE) is used to choose the best parameters during iterations. RMSE is calculated as follows:

$$E = \sqrt{\frac{1}{n}\sum_{i:1}^{n}|A_i - F_i|^2} \qquad \text{(Eq. S8)}$$

In the above equation, A is the experimental DTG data and F is the composite model data for n observations.

The calculations start by selecting initial values as well as lower and upper bounds for A_j, E_j and $x_{j,0}$. $x_{j,0}$ is the fraction of the component in the biomass ($\frac{\alpha_{j,0}}{\alpha_0}$). First, conversion curves are constructed using A_j, E_j for each component. During this process, the temperature integral is evaluated numerically for each data point. Conversion curves are differentiated and the DTG curves for the model are obtained for each component. Next, the DTG curves are rescaled using $x_{j,0}$ for each component and added up to compare with the experimental DTG curve. Root Mean Squared Error is used to evaluate the precision of the fit of the model during the iteration and choose the better dataset inside the iterations.

Results

The calculated kinetic parameters are presented in Table S1, and the pseudocomponent compositions are provided in Table S2. TGA results, along with model fits to the experimental DTG data for the three feedstocks, are presented in Figure S1.

Table S1. Kinetic Parameters (C: Cellulose, H: Hemicellulose, L: Lignin)

	**Kinetic Parameter**	**Pseudocomponent**						**R^2**
		**C**	**H1**	**H2**	**L1**	**L2**	**L3**	
Olive Pit	E(kJ/mol)	230	90	167.5	52	46	80	0.960
	A(min^{-1})	$8x10^{21}$	$1.8x10^{10}$	$2x10^{18}$	$2x10^{4}$	$2.2x10^{25}$	10^{7}	
Reed Pellet	E(kJ/mol)	195	90	167.5	48	40	-	0.993
	A(min^{-1})	$9x10^{18}$	$8x10^{9}$	$1.4x10^{18}$	$1.15x10^{4}$	$2.4x10^{4}$	-	
Wood Chips	E(kJ/mol)	220	98	80	58	40	-	0.991
	A(min^{-1})	$5.8x10^{20}$	$8x10^{10}$	$0.6x10^{7}$	$3.15x10^{4}$	$5x10^{4}$	-	

Table S2. Pseudocomponent Compositions (C: Cellulose, H: Hemicellulose, L: Lignin)

	**Pseudocomponent**						
	C	H1	H2	L1	L2	L3	Moisture
**Olive Pit**	0.3	0.12	0.16	0.11	0.18	0.06	0.07
**Reed Pellet**	0.3	0.15	0.16	0.16	0.165	-	0.07
**Wood Chips**	0.33	0.16	0.08	0.14	0.22	-	0.07

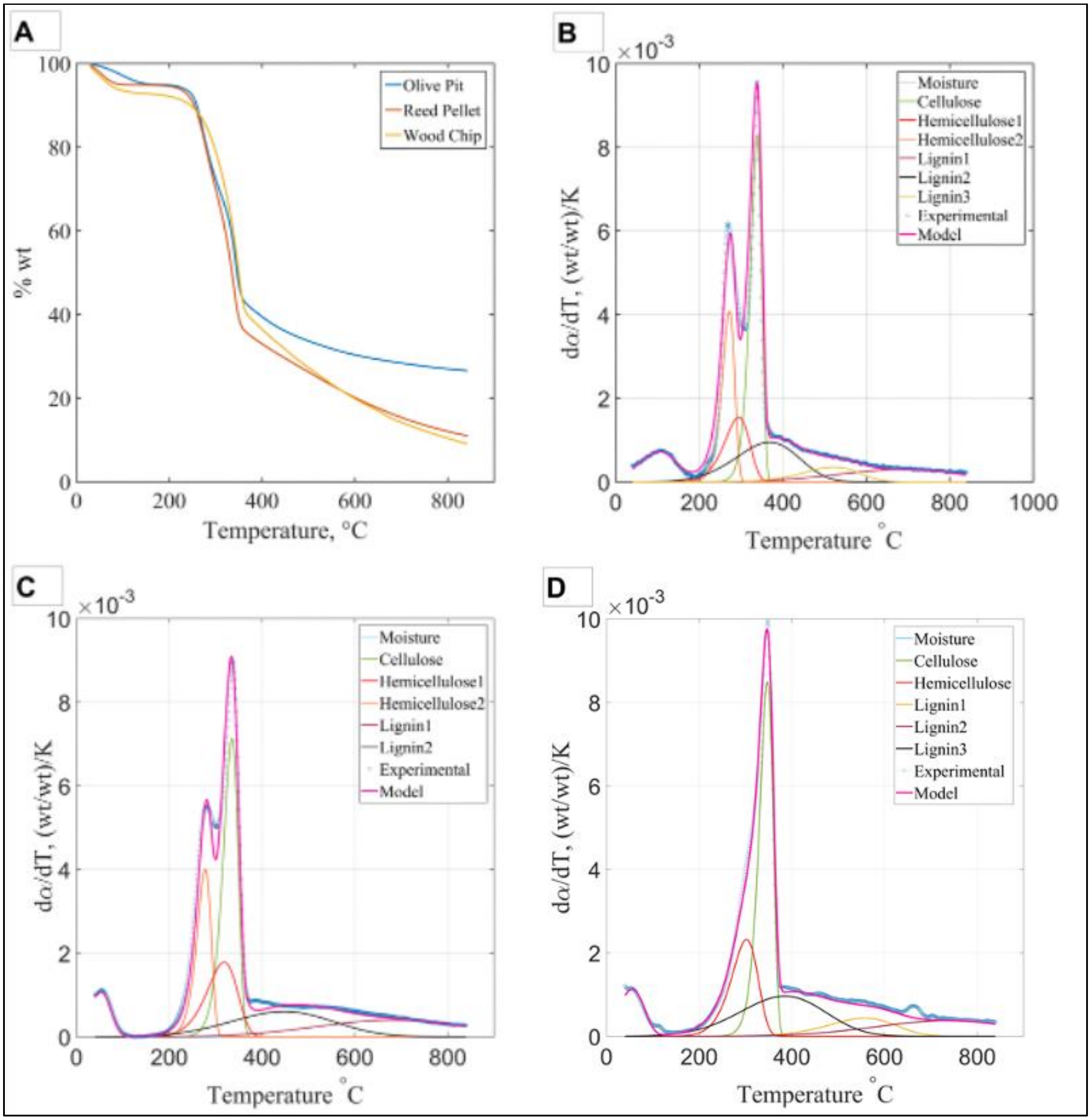

Figure S1. A) TG curves for three different feedstocks. Experimental DTG data with model fits for B) olive pit, C) reed pellet, and D) wood chips.
